# Expression Profiles of Integrin-Linked Kinase, Vascular Endothelial Growth Factor A, and Ephrin Type-A Receptor 2 in Colorectal Cancer Lymph Nodes

**DOI:** 10.7759/cureus.71242

**Published:** 2024-10-11

**Authors:** Hayat Ahmed, Hazhmat Ali, Kareem M Ahmed

**Affiliations:** 1 College of Medicine, University of Duhok, Duhok, IRQ

**Keywords:** cancer metastasis, colorectal cancer, ephrin type-a receptor 2, integrin-linked kinase, vascular endothelial growth factor

## Abstract

Background

Colorectal cancer (CRC) is the third most frequently diagnosed cancer globally, with a high incidence of morbidity and mortality. Early diagnosis of CRC is crucial for determining appropriate treatment regimens, thereby prolonging overall survival rates and improving prognostic outcomes.

Objectives

This study aimed to investigate the expression profiles of specific proteins in the lymph nodes (LNs) of CRC patients, including integrin-linked kinase (ILK), vascular endothelial growth factor A (VEGFA), and ephrin type-A receptor 2 (EphA2), through immunohistochemical studies.

Methods

The study involved a sample of 76 patients clinically diagnosed with CRC who were referred by their specialist oncologist. Tumor staging was determined based on the TNM classification. Immunohistochemical studies were conducted to measure the expression patterns of the candidate markers (ILK, EphA2, and VEGFA). Expression levels were scored as negative, low, or high.

Results

The highest percentage of CRC patients were diagnosed with conventional adenocarcinoma, predominantly in stages II and III. Of the 76 CRC tissue specimens, the majority (46, 60.52%) tested negative for lymphatic invasion, while the remaining 30 (39.47%) tested positive. According to the TNM classification, 14 samples had N1 (one invaded LN), and 16 had N2 (two or more invaded LNs). Furthermore, the percentage of patients with low and high EphA2 expression was significantly higher (p<0.0001) compared to the negative expression controls. Regarding ILK, 15 cases (50.0%) showed negative expression, while an equal number displayed positive expression. Additionally, the group with low VEGFA expression was statistically significantly higher (p=0.01) compared to the negative control.

Conclusion

The expression levels of EphA2, ILK, and VEGFA were found to be higher in LNs with lymphatic invasion compared to those with negative expression, highlighting the role of these proteins in CRC progression.

## Introduction

Colorectal cancer (CRC) is the third most frequently diagnosed type of cancer globally, with a high incidence of morbidity and mortality. In Asia alone, the incident rate dramatically increased in 2018, with a mortality rate of 52.4% per 100,000 population, regardless of age and gender. This substantial rise could be attributed to various risk factors, including dietary lifestyle, aging population, smoking, and sedentary life. Based on the emerging epidemiological data, some Asian countries, such as Japan, Singapore, and South Korea, have conducted nationwide screening programs that are effective in the early detection and management of CRC [1]. The majority of CRC cases are localized either with or without lymph node (LN) metastasis. However, approximately 20% of cases are diagnosed with the metastatic form of the disease, mainly in the liver. Despite the emergence of a wide range of anticancer drug categories, the surgical recession is still considered the golden standard for localized tumors. For patients with LN metastases, conventional chemotherapy with adjuvant therapeutics is usually recommended. Concerning metastatic or recurrent CRC, chemotherapy remains the main option, together with radiotherapy and surgery if deemed necessary [2].

A comprehensive understanding of the molecular biology of CRC is a crucial step toward anticancer drug design. So far, a diverse range of molecules have been developed to combat this disease, including immunotherapy and targeted therapy. One of the common examples is the anti-EGFR (monoclonal antibodies targeting epidermal growth factor receptor) agents, which have been proven effective in metastatic CRC [3]. Despite the substantial development in the field of anticancer therapeutics, multi-drug resistance still remains an immense challenge [4]. Previous research published by our institution focused on the molecular mechanisms underlying the pathogenesis of CRC. It was shown that a wide range of proteins, mainly matricellular proteins, are responsible for promoting cell migration and invasion, thus inducing epithelial-mesenchymal transition (EMT), the key player that initiates the metastatic cascade [5]. Others reported that certain gene mutations, such as KRAS, BRAF, and p53, play a critical role in CRC metastasis, which could be regarded as potential biomarkers and therapeutic targets as well [6]. Furthermore, the overall survival rate was shown to be extended from 5.9 to 9.3 months when the combination therapy (chemotherapy with targeted therapy) was administered to patients with metastatic CRC [7].

Early detection and diagnosis of CRC are considered a key feature in determining appropriate treatment regimens for patients, prolonging the survival rate and improving the prognostic outcomes. The present study aims to investigate the expression profiles of certain protein molecules in LNs of a sample of CRC patients, including integrin-linked kinase (ILK), vascular endothelial growth factor A (VEGFA), and ephrin type-A receptor 2 (EphA2), using immunohistochemical studies.

## Materials and methods

Study design and ethical considerations

This cross-sectional study was initially approved by the Research Ethics Committee, College of Medicine, University of Duhok (reference number: 13072022-7-2). The current study involved a sample of 76 patients clinically diagnosed with CRC who had been referred by their oncologist to the Central Laboratory Department of the General Directorate of Health in Duhok Province. The study began with the sample collection phase on the 18th of July 2022 and was completed on the 20th of December 2023 for a total duration of one year and five months. Participants above 18 years who were diagnosed with CRC regardless of tumor stage and gender were included in this study. However, patients who refused to participate in the current study and did not provide consent were excluded. A consent form was provided to each patient indicating their approval to be included in the current study. Upon their consent, necessary information regarding demographic characteristics (age, gender, etc.) and other relevant clinical data was taken from their pathological report. Then, their tissue specimens, including the already examined slides and paraffin-embedded blocks, were taken for further investigations by the research team. Any sample with a scanty amount of tissues or without LNs was excluded from this study.

TNM staging 

Once the sample collection phase was completed, all specimens were re-examined by authors supervised by a pathologist to ensure accuracy and avoid discrepancy. Some of the specimens were stained again (if necessary) using the conventional hematoxylin and eosin (H&E) staining protocol [8]. Upon confirming the appropriate cases for the study, another round of examination was conducted to determine the tumor staging of CRC using the TNM classification. It involves staging cancer based on three criteria: tumor size (T) and its spread into nearby tissues; the existence of nodal metastasis (N); and distant metastasis (M); tumor spearing into distant organs such as the liver and lungs. Although recent updates have been introduced by the 8th edition of the American Joints Committee on Cancer (AJCC), the basic staging structure remained the same [9]. Because the current study focused on examining the expression pattern of ILK, EphA2, and VEGFA in LNs, all other samples with no lymphatic invasion were excluded, and only the samples with positive lymphatic invasion were chosen for further immunohistochemical experiments.

Immunohistochemistry

The histopathological specimens were examined for appropriate selection of malignant tissues, including LNs. Then, the corresponding block (positive for LN invasion) was pointed out for further immunohistochemical studies. For that purpose, a 4 µm thick section from each sample was obtained for the immunohistochemistry (IHC) staining of the candidate markers investigated in our study (ILK, EphA2, and VEGFA), which was carried out against both positive and negative controls. The Avidin Bionin Complex (ABC) detection system was carried out for the staining protocol using the automated IHC staining method. Once the slides were ready for interpretation, the IHC results were examined by two certified pathologists autonomously without providing patients with clinical features to ensure obtaining accurate results. For IHC scoring, 10 powered fields were randomly selected, and approximately 100 cells were counted per field. The recorded score is based on both the percentage of immunoreactive cells and the intensity of staining to obtain the overall score [10]. After recording the final score for each sample, the results were classified into three main categories: negative, low, and high expression, respectively.

Statistical analysis

The statistical analysis of the clinical features of CRC with the obtained IHC results was carried out using Statistical Package of Social Science (SPSS) version 26. Descriptive statistics were used to calculate the frequency and percentages of the demographic characteristics and the clinical features of CRC. All variables were expressed as numbers and percentages. Dunnett's test was used to compare different variable groups mainly low expression, and high expression with the negative expression. The probability (p-value) was used to determine the statistical significance between variables. The p-values of less than 0.05 were considered statistically significant.

## Results

Demographic characteristics and clinical features of CRC patients

The demographic characteristics and the clinical features of CRC are summarized in Table 1. A total of 76 CRC patients were recruited for the current study, with an age range of 20-70 years. Regarding gender, the number of females (41, 53.94%) was higher than males (35, 46.05%). The majority of participants were diagnosed with conventional adenocarcinoma (66, 86.84%), followed by mucinous adenocarcinoma (8, 10.52%) and signet ring cell (2, 2.63%), respectively. Based on the TNM staging, the higher percentages fell under stages II (30, 39.47%) and III (22, 28.94%), followed by stage I (19, 25%), and finally stage IV (5, 6.57%).

**Table 1:** Clinical features of CRC patients CRC, colorectal cancer

Variables	Number	Percentage (%)
Age (above 18 years)	76	100%
Male	35	46.05
Female	41	53.94
Conventional adenocarcinoma	66	86.84
Mucinous adenocarcinoma	8	10.52
Signet ring cell	2	2.63
Stage I	19	25
Stage II	30	39.47
Stage III	22	28.94
Stage IV	5	6.57

Clinical features of lymph nodes

The total number and percentage of LNs isolated from 30 CRC tissue specimens are illustrated in Table 2. From a total of 76 CRC tissue specimens, the majority (46, 60.52%) were negative for lymphatic invasion, whereas the remaining (30, 39.47%) were positive. Based on the TNF classification, 14 samples (16.09%) had N1 (one invaded LN) and 16 (18.39%) had N2 (two or more invaded LN). Samples with LN invasion were used for further experiments. 

**Table 2:** Clinical features of LNs in CRC patients based on TNM staging CRC, colorectal cancer; LN, lymph node

Variables	Number	Percentage (%)
No LN metastasis	46	60.52
LN metastasis	30	39.47
N0	57	65.52
N1	14	16.09
N2	16	18.39

**Figure 1:**
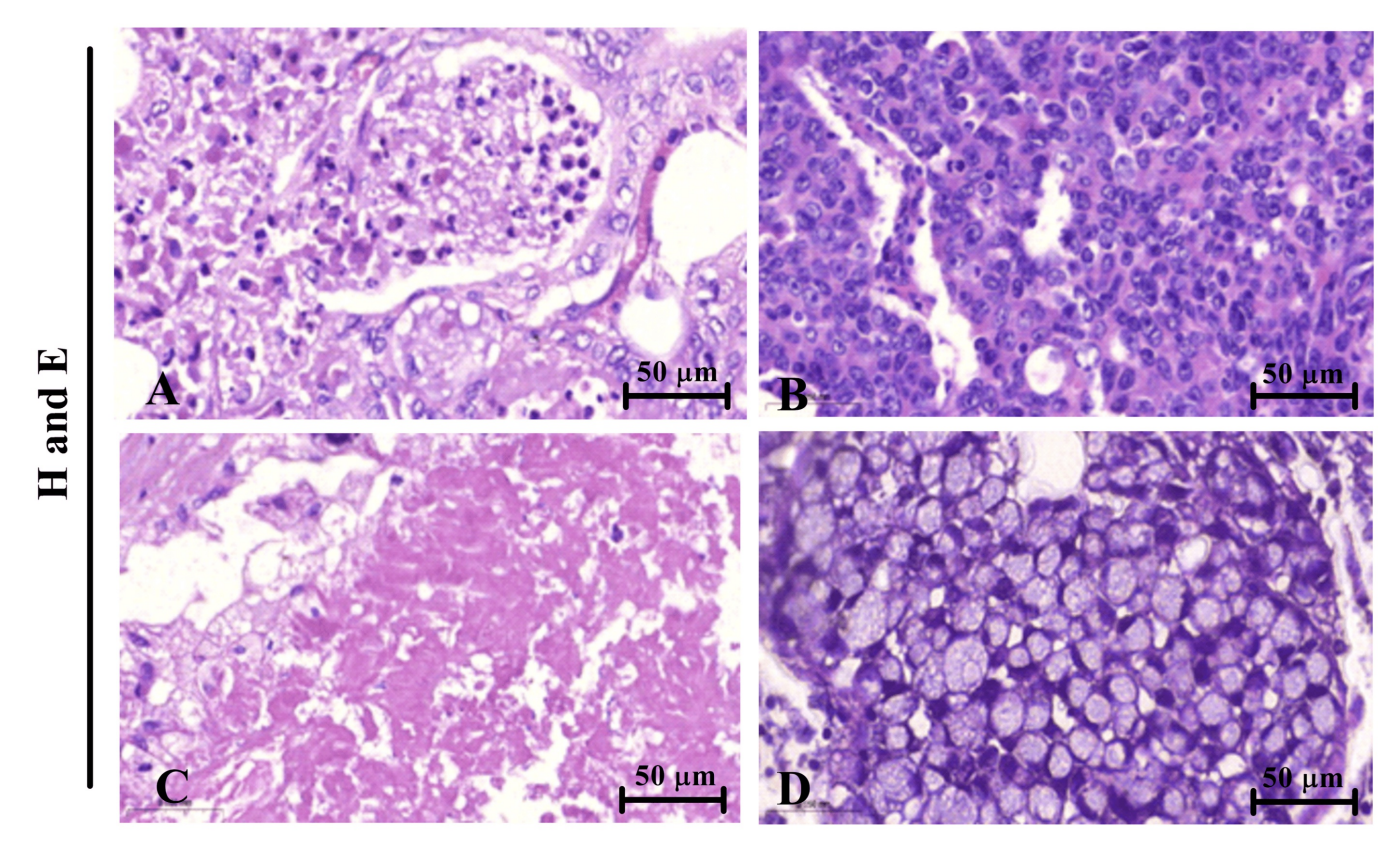
LNs with lymphatic invasion Histopathological representative images (H&E staining) showing the different types of CRC. Colorectal adenocarcinoma (A and B) with LN positive lymphocytic infiltration, x40. Mucinous adenocarcinomas (C), x40, followed by signet ring cell, x40 (D). CRC, colorectal cancer; LN, lymph node

To further explore the invaded LNs in different types of CRC tissues, histopathological specimens were stained with the conventional H&E. As illustrated in Figure 1A and Figure 1B, colorectal adenocarcinomas are observed, characterized by LN-positive lymphocytic infiltration followed by mucinous adenocarcinomas (Figure 1C) and signet ring cell (Figure 1D), respectively.

EphA2 expression in lymph nodes of CRC patients 

Immunohistochemical studies were performed to determine the expression pattern of EphA2 in LNs of CRC patients. Results indicated that the expression was high in 14 cases (46.67%) and low expression was detected in 13 specimens (43.33%) with three cases of negative expression (10%) (Table 3).

**Table 3:** Frequency and percentage of EphA2 expression in CRC LNs CRC, colorectal cancer; LN, lymph node; EphA2, ephrin type-A receptor 2

EphA2 expression pattern	Frequency (no. 30)	Percentage (%)
High expression	14	46.67
Low expression	13	43.33
Negative expression	3	10.00

**Figure 2:**
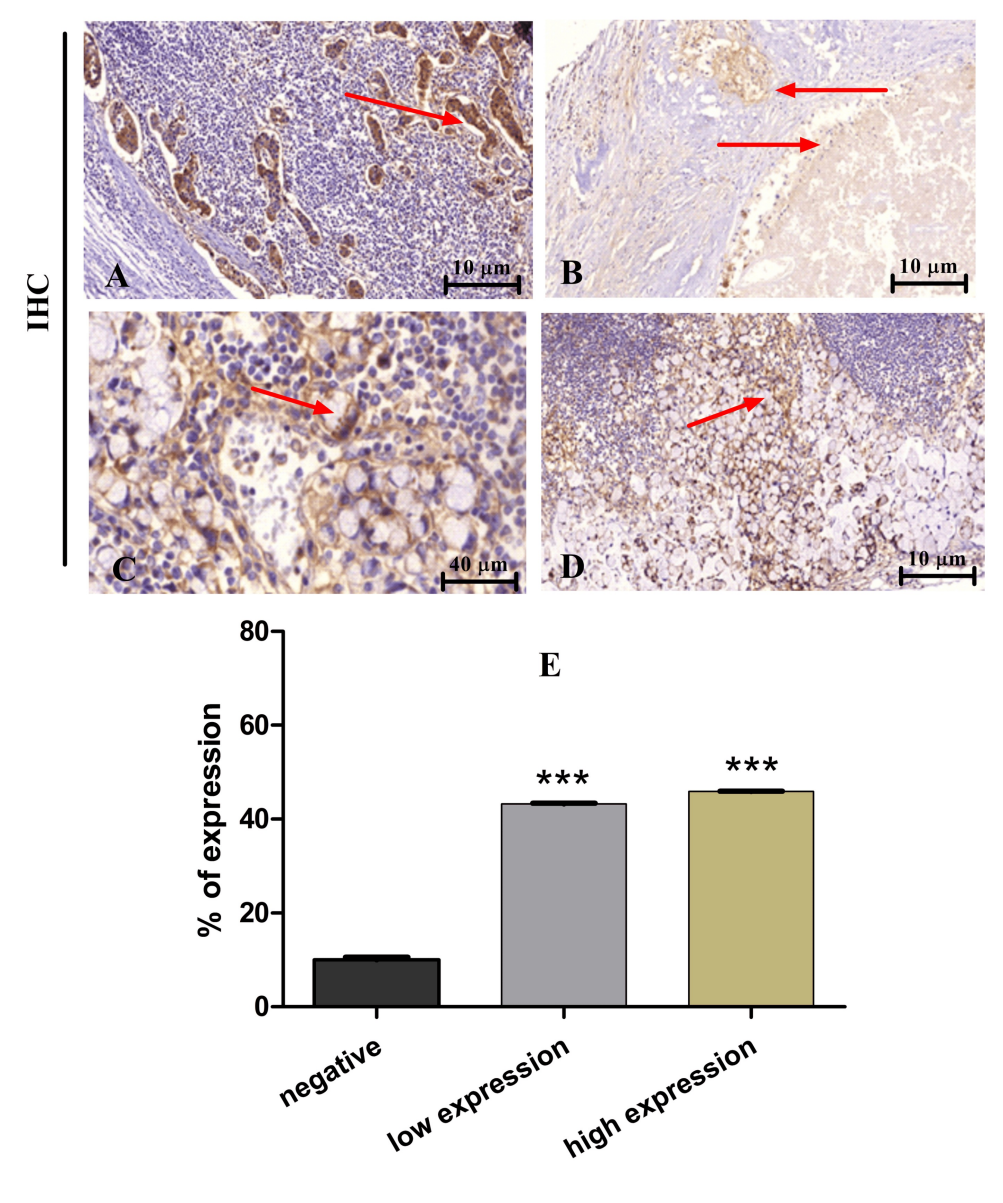
EphA2 expression in CRC LNs (A) High expression, x10. (B) Mucinous low expression, x10. (C and D) High expression signet ring cell, x40 and x10, respectively. The statistical differences between negative, low, and high expression (E). ***p < 0.0001 CRC, colorectal cancer; LN, lymph node; EphA2, ephrin type-A receptor 2

Furthermore, immunohistochemical images from the stained LNs showed the same expression pattern of EphA2. Representative images in Figure 2 show high expression (Figure 2A) and mucinous low expression ( Figure 2B). On the other hand, Figure 2C and Figure 2D represent high-expression signet ring cells of x10 and x40, respectively. Moreover, patients’ groups with low and high EphA2 expression were significantly higher (p<0.0001) compared to negative expression controls (Figure 2E). 

ILK expression in lymph nodes of CRC patients

The ILK expression in LNs of CRC patients is characterized in Table 4. Based on the results obtained, 15 cases (50.0%) showed negative expression, and an equal number displayed positive expression. Further analysis of the samples with positive expression indicated that only three (10%) samples showed high expression compared to 12 cases (40%) with low expression.

**Table 4:** Frequency and percentage of ILK expression in CRC LNs CRC, colorectal cancer; LN, lymph node; ILK, integrin-linked kinase

ILK expression pattern	Frequency (no. 30)	Percentage (%)
High expression	3	10
Low expression	12	40
Negative expression	15	50

**Figure 3:**
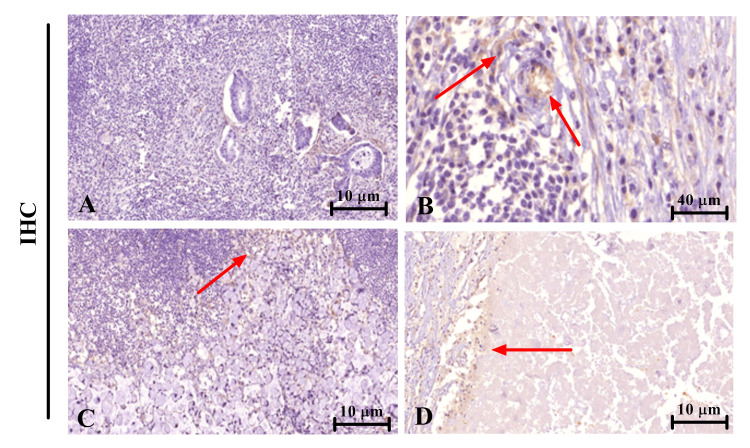
ILK expression in CRC LNs (A) Negative expression, x10. (B) Low expression, x40. (C and D) Low expression signet ring cell, x10, and mucinous low expression, x10. CRC, colorectal cancer; LN, lymph node; ILK, integrin-linked kinase

Figure 3 displays the immunohistochemical imaging of ILK expression in LNs. The negative expression (Figure 3A), low expression (Figure 3B), low expression signet ring cell (Figure 3C), followed by a mucinous low expression (Figure 3D).

VEGFA expression pattern in lymph nodes

The present study also examined the expression pattern of VEGFA in LNs. Table 5 shows that no high expression was detected in the examined specimens compared to 18 cases (60%) that indicated low expression. The other 12 cases (40%) displayed no expression for the aforementioned marker.

**Table 5:** Frequency and percentage of VEGFA expression in CRC LNs VEGFA, vascular endothelial growth factor A; CRC, colorectal cancer; LN, lymph node

VEGFA expression pattern	Frequency (no. 30)	Percentage (%)
High expression	0	0
Low expression	18	60
Negative expression	12	40

**Figure 4:**
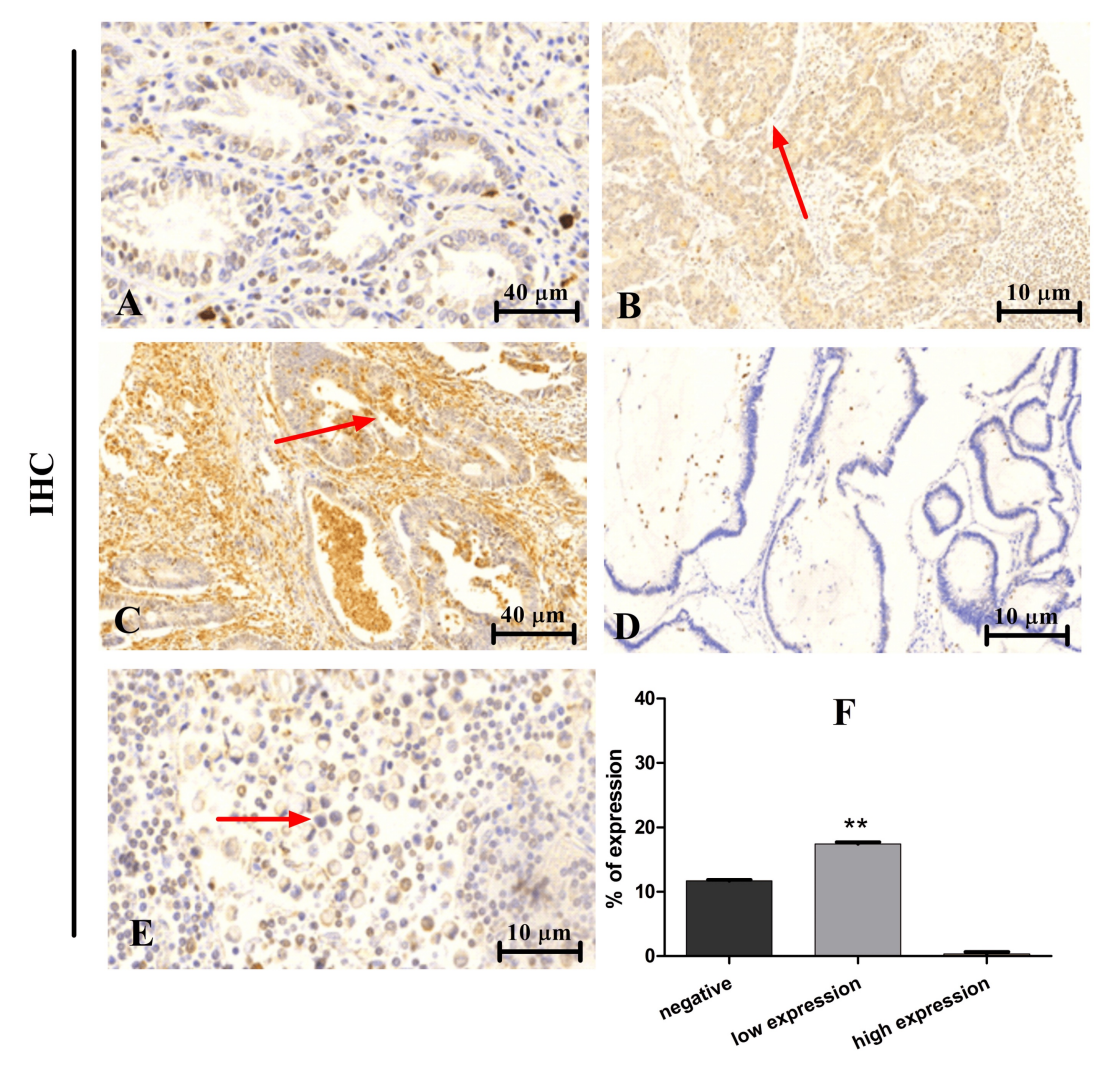
VEGFA expression in LNs (A) Negative expression, x40. (B) Low expression, x10. (C) High expression, x40. (D) Mucinous negative expression, x10. (E) Low expression, x10, with signet ring cell. The statistical differences between negative and low expression groups (F). **p<0.01 VEGFA, vascular endothelial growth factor A; LN, lymph node

Representative images of VEGFA expression by IHC are seen in Figure 4. The first image shows negative expression (Figure 4A), low expression (Figure 4B), followed by high expression AD (Figure 4C), mucinous negative expression (Figure 4D), and low expression with signet ring cell (Figure 4E), respectively. Moreover, the group with low expression was statistically significantly higher (p=0.01) compared to the negative control (Figure 4F). 

## Discussion

A thorough understanding of the pathophysiological features of CRC has made a wide range of treatment options available, from surgical tumor recession to targeted therapy and immunotherapy. This has significantly prolonged the overall survival rate. Early detection of the disease before the metastatic form manifests is considered one of the key elements in overcoming this kind of cancer [11]. While numerous studies have explored the expression levels of different molecules in tumor tissues, the current study aims to investigate the expression pattern of certain proteins (ILK, EphA2, and VEGFA) in a sample of LNs from CRC.

EphA2 is a glycoprotein receptor that belongs to the Ephrin family, the largest receptor family of tyrosine kinases (RTK). Based on the structural homology and affinity, the Eph family of receptors is divided into two subclasses: EphA and EphB. Among the diverse types of receptors within the Eph family, the EphA2 transformation of normal cells into malignant, angiogenesis, and metastasis has been comprehensively studied in many types of cancers, such as melanoma, glioblastoma, and breast cancer [12]. The current study’s findings showed that the frequency and the percentage of EphA2 expression in colorectal LNs were substantially higher compared to negative expression. Our findings are supported by earlier studies concluding that the EphA2 expression in CRC is associated with cell migration and invasion. It is also considered a poor prognostic marker, particularly in stages II/III, providing insights for further investigations of EphA2 as a novel prognostic marker [13]. Since it is detected in the early stage of CRC, the diagnostic potential of EphA2 has been extensively examined. A study investigating the diagnostic value of EphA2 reported that the elevated serum EphA2 level in CRC patients was significantly higher compared to the control, with an overall sensitivity of 60.40% and a specificity of 92.8% [14]. Numerous preclinical studies have focused on EphA2 as a promising therapeutic target in cancer, particularly in CRC. It was shown that targeting EphA2 by the tyrosine kinase inhibitor NVP-BHG712 and its derivatives substantially reduced cell proliferation and induced programmed cell death (apoptosis) in CRC cell lines [15]. Despite its selectivity against EphA2, it seems difficult to design a molecule targeting all other receptors since many tumors express different kinds of type A and B receptors.

ILK is a serine/threonine protein kinase that was found to play a pivotal role in the adhesion between cells and matrix, particularly during carcinogenesis [16]. Our results concluded that ILK expression was detected in 50% of the histopathological specimens. Such findings are consistent with recent studies indicating that ILK expression is one of the hallmarks of the pathogenesis of CRC. Its overexpression was found to be correlated with cancer progression, poor prognosis, and development of chemo-resistance, particularly in lung cancer and glioma. Moreover, the overexpression is related to advanced stage and high tumor grade, which might be considered a prognostic marker of metastasis in LNs [17]. The possible mechanism behind the critical role of ILK in cancer progression could be due to its EMT-inducing effects with altered expression in epithelial markers, β-catenin and E-cadherin [18]. Studies examining the therapeutic potential of ILK emphasized that targeting ILK could provide synergistic effects epically when used with the currently available chemotherapeutic agents in various types of cancer. It may also substantially reduce the chemo-resistance in gastric cancer [19].

Finally, we investigated the expression pattern of VEGFA in this study. VEGFs are primary growth factors that play an essential role in angiogenesis. Upon binding to their corresponding receptors, they promote the proliferation and migration of endothelial cells and vascular endothelial cell survival [20]. The VEGFs belong to the platelet-derived growth factor (PDGF) family that regulates various cellular pathways involved in cell proliferation and survival, thus maintaining the structural integrity of the cell [21]. Although no single sample showed a high expression, the number and percentage of specimens with VEGFA low expression were considerably higher compared to negative expression. These findings are consistent with recent studies highlighting the correlation of VEGF expression with alterations in cell morphology and EMT. Targeting the VEGF signaling pathway was shown to significantly reduce CRC cell migration and invasion [22]. Due to its essential contribution to carcinogenesis, VEGF has been a rational target in anticancer therapy. Although several agents have been designed to target VEGF signaling pathways, only bevacizumab has been approved by the FDA as a suitable option for the advanced form of CRC in combination with conventional chemotherapy [23]. Another tyrosine kinase inhibitor (Vatalanib) was shown to inhibit VEGF-mediated angiogenesis, thus reducing tumor growth and metastasis by blocking VEGFR-1, VEGFR-2, and VEGFR-3 [24]. Other cell-based studies concluded that targeting VEGF by siRNA is not only effective in reducing metastatic cascade but also increasing the sensitivity of the chemotherapeutic agents [25].

Despite the diligent efforts by researchers to conduct the current study, some possible limitations can be noticed. Among the 76 collected CRC specimens, only 30 samples with positive lymphatic invasion were selected for further experiments. This is due to the fact that the clinical sample collection and obtaining patient consent are considered quite a challenging task. Furthermore, the expression pattern of the proteins examined in the current study was done using IHC alone. Future research should utilize other available sophisticated methods to determine these proteins at mRNA and protein levels, such as polymerase chain reaction (PCR) and western blot, respectively.

## Conclusions

In conclusion, we have, for the first time in our region, demonstrated the expression profiles of certain molecules in CRC LNs. The expression levels of EphA2, ILK, and VEGFA were found to be higher in LNs with lymphatic invasion compared to those with negative expression. These findings collectively highlight the role of these proteins in the progression of CRC. Future studies should focus on exploring additional mechanisms for targeting these proteins to increase the sensitivity of chemotherapeutics, thereby improving overall patient survival rates.
